# Unlocking the potential of bacteriophage-based therapeutic gene delivery in hepatocellular carcinoma

**DOI:** 10.1186/s43046-026-00406-2

**Published:** 2026-09-04

**Authors:** Sreemoyee Mitra, Damini, Mahima Devi, Sreyosi Guha Niyogi, Utkarsh Pratap Singh, Debanjan Lo, Hitesh Chandra Bishtania, Shubham Thakur

**Affiliations:** Department of Pharmaceutics, ISF College of Pharmacy, Moga, India

**Keywords:** Hepatocellular Carcinoma, Oncology, Bacteriophages, Genome Editing, Immunotherapies, Phage Vectors, CRISPR-Cas systems, Gene Delivery

## Abstract

Liver cancer, mainly hepatocellular carcinoma (HCC), remains a global health burden marked by poor prognosis with limited therapeutic efficacy, and high recurrence rates. HCC remains one of the most lethal malignancies worldwide, with limited therapeutic options and high resistance to conventional treatments. Despite low therapeutic efficacy, molecular heterogeneity, treatment resistance and high recurrence rate, hepatocellular carcinoma (HCC) is still a significant health problem worldwide. These restrictions have stimulated the research of focused methods for delivering therapeutic genetic payload into cancer cells. Bacteriophages have been gaining growing attention as an emerging delivery platform due to their genetic versatility, ease of engineering, ability to be surface modified and payload targeted. In this narrative review, the therapeutic potential of engineered bacteriophages in the context of HCC therapy is critically analyzed focusing on phage display-mediated tumor targeting, phage-mediated intracellular gene delivery, TRAIL gene delivery, and CRISPR/Cas-based therapeutic strategies. It has been previously noted in the literature that phage display can be used to attach tumor-targeting ligands to the surface of a phage, which may aid in the recognition of receptors at the tumor site and promote targeted delivery to the receptor. Therapeutic application is stunted by inefficient trafficking to the cytosol, endosomal degradation, immune recognition and clearance, vector stability, manufacturing scalability and regulatory issues. In conclusion, engineered bacteriophages are a promising and versatile tool for targeted gene delivery in HCC but more mechanistic, preclinical and translational research is needed to prove their therapeutic effectiveness and clinical usefulness for this purpose.

**Graphical Abstract:**

**Figure:**
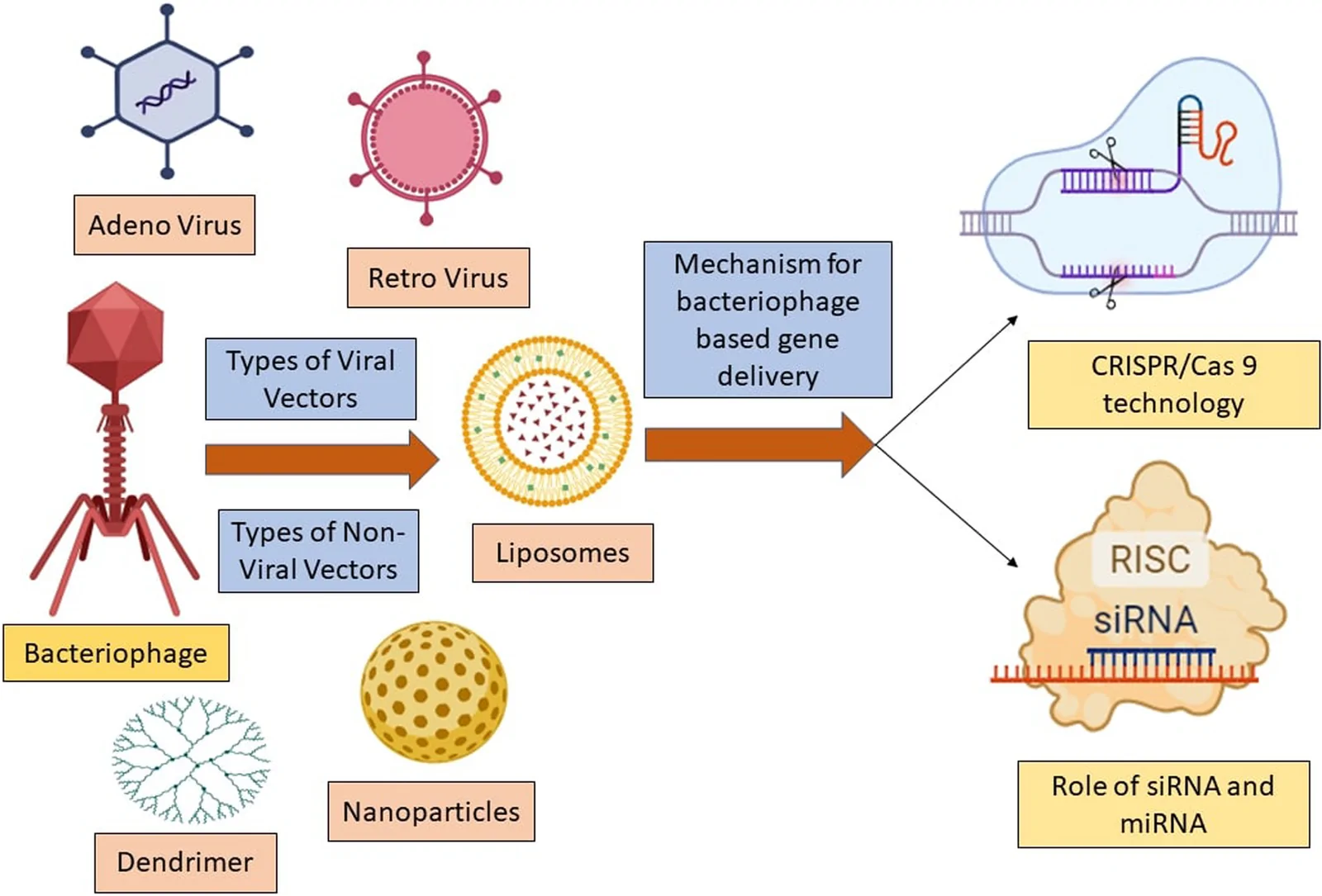

## Introduction

 Hepatocellular carcinoma (HCC) is the most common primary malignant liver tumor and a serious health problem all over the world. The latest estimates from the International Agency for Research on Cancer (IARC) indicate that liver cancer is responsible for the deaths of over 730,000 people and leads to the diagnosis of some 850,000 new cases worldwide in 2024. Liver cancer was one of the major causes of cancer deaths worldwide, due to its high mortality burden. The burden of liver cancer diseases is further likely to rise in the next few decades due to chronic viral hepatitis, alcohol-related liver disease, metabolic dysfunction-associated steatotic liver disease, obesity, and other metabolic risk factors [1]. As chemotherapy does not enhance survival rates, current therapies consist on tumour removal and liver transplantation [2]. Patients in advanced stages are ineligible for surgery or transplantation owing to metastases and need to turn to nonsurgical interventions, including transarterial embolisation, transarterial chemoembolization, and ablation treatment [2]. HCC is a markedly diverse malignancy, as revealed by current high-throughput sequencing and gene expression profiling at both the molecular and histological levels. Conventional treatment modalities such as surgery, ablation, chemoembolization, systemic chemotherapy, and molecularly targeted medicines have partial success for hepatocellular carcinoma (HCC); however, they are inadequate for advanced-stage HCC regarding their effectiveness. The efficacy of chemotherapy in hepatocellular carcinoma (HCC) is limited by chemoresistance and systemic adverse effects. To enhance the efficiency and safety of chemotherapeutics in hepatocellular carcinoma therapy, targeted carriers such nanoparticles have undergone efficacy testing in fundamental research, but remain inadequate for clinical use [3]. Recent advancements in molecular targeted agents (MTA) have illuminated treatment for hepatocellular carcinoma (HCC), taking account of the molecular expression variances inside the tumour.

Although surgical, locoregional and systemic therapies have improved in the treatment of advanced HCC, there remains the problem of heterogeneity, resistance, immune evasion and toxicity of the tumours, emphasising the need for more targeted molecular therapies [4, 5]. To address the challenges of current viral and non-viral vectors, engineered bacteriophages with targeting ligands and therapeutic genetic material have been developed and genetically engineered or selected to deliver more precisely in HCC, as they are less immunogenic and have higher capacity, and are easier to manufacture [6, 7].

The most prevalent kinds of biological entities in the biosphere are bacteriophages, which are obligatory parasites of bacteria [8]. Bacteriophage molecular machinery of host cells to express their personal genes because their genomes are entirely devoid of the genes required for autonomous reproduction. Bacteriophages are a prospective tool in several biotechnology disciplines because of their special qualities [9]. Further, the emergence and development of genetic engineering have been significantly influenced by bacteriophages and anti-phage defence mechanisms.

Even with all of the progress made in clinical medicine, ailments still afflict contemporary scientists. Up to one in five people worldwide will have cancer at some point in their lives, making it one of the most difficult of these illnesses [10]. Researchers are seeking new therapies that can address issues like limited accessibility, adverse side effects, insufficient effectiveness, and high costs. Phage-based therapy is an example of a new and emerging treatment approach. Given its considerable potential to develop into a dependable cancer treatment, this relatively new use of bacteriophages is a fascinating line of inquiry and is currently being investigated further [11]. They have reported that these phages have the potential to combat the hepatic cancer due to their structure, biology, which was proven to be safe as per the various clinical measures. Further, it has been reported that these are use in photodynamic malignance therapy of hepatic cancer [12] due their efficacy in the targeted expression of transgenes from modified prokaryotic viruses in tumor cells, which is the coined to be the main emphasis of the current study. Additionally, this review emphasizes that how these bacteriophages are genetically transferred, along with their therapeutic uses.

## Types of vectors involved in gene delivery

**Fig. 1:**
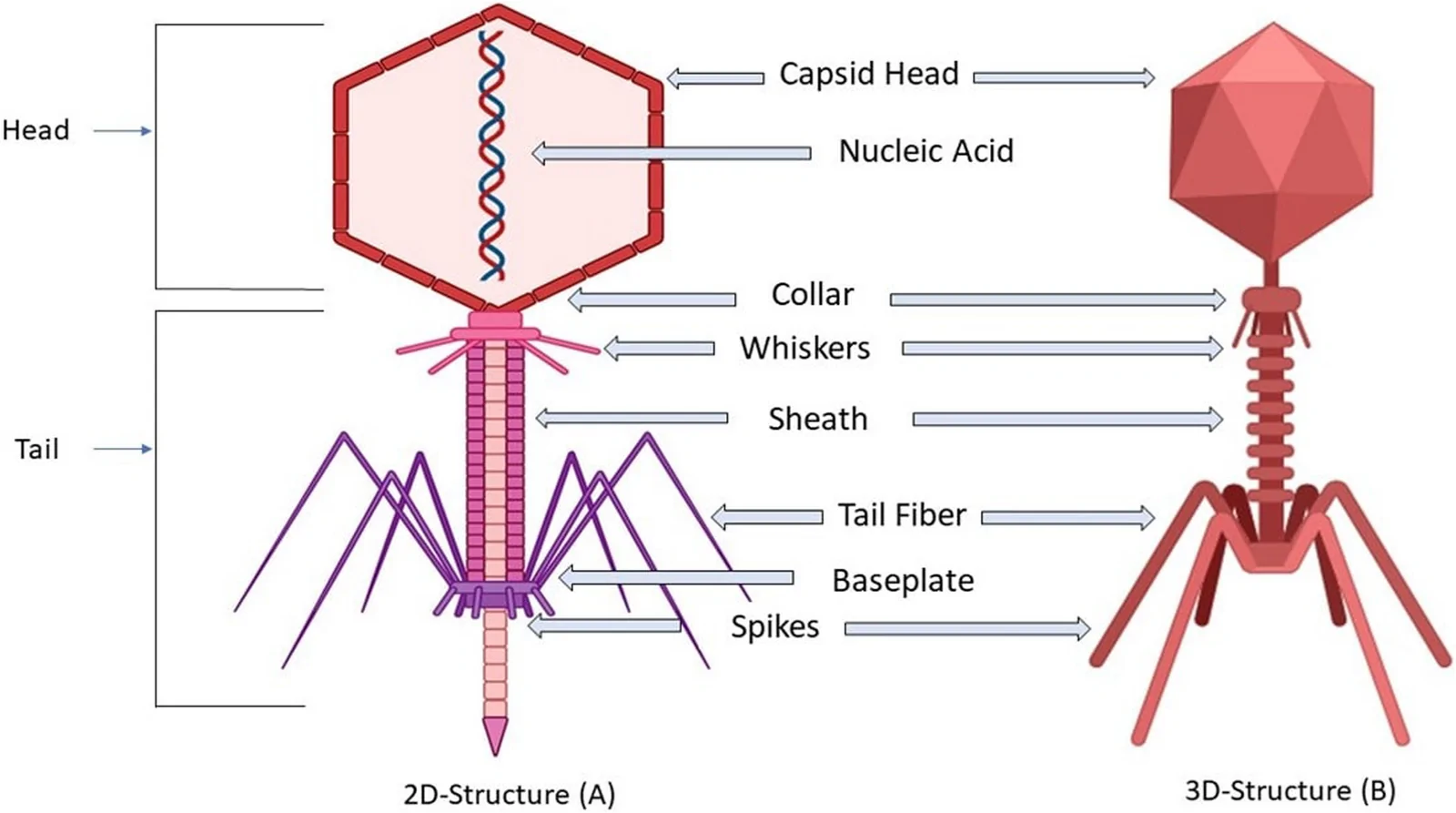
Structure of Bacteriophages: **A**: 2- Dimensional; **B**: 3-Dimensional

The bacteriophages are majorly composed of a head and a tail as illustrated in Fig. 1 [13]. The virus’s genetic substance, single stranded or double stranded deoxyribonucleic acid (DNA) or ribonucleic acid (RNA), is in the head that are mainly enclosed in the capsid. While the baseplate at the end of the bacteriophage’s tail, which develop the thin and long fibrils, enables the phage adhere to the bacteria, which counts as an essential element of bacteriophage [14]. Further, the receptor-binding proteins in the baseplate works in identification of chemicals on the bacterial membrane’s surface. Through this way, it interacts with bacterial cells in a highly precise way via the targeted binding that improves the compatibility of the membrane with receptors.

**Fig. 2:**
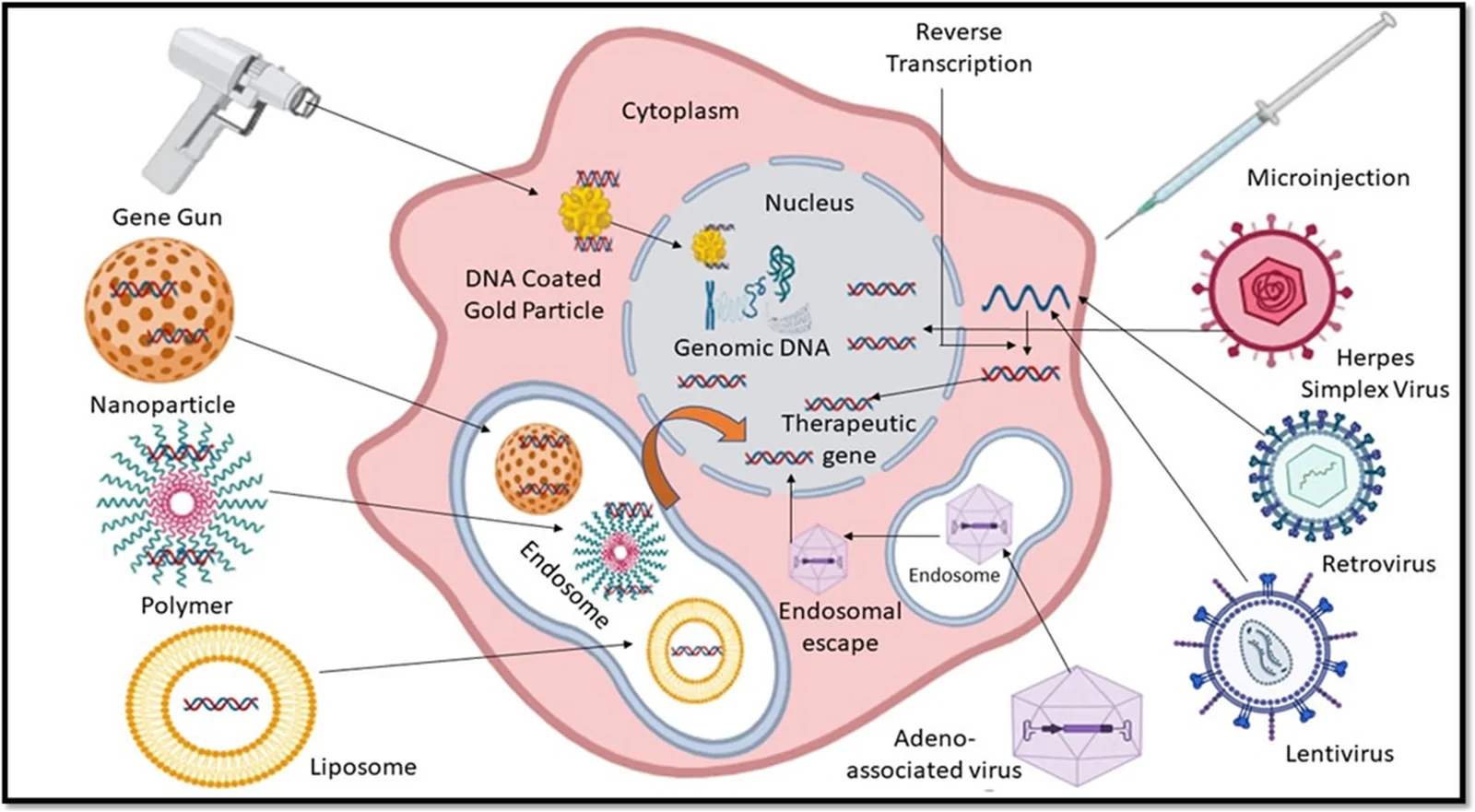
Schematic representation of targeted gene therapy for hepatic cancer with viral and non-viral vectors

Gene delivery vectors are the powerful tools that are used to introduce genetic material into the cells for therapeutic and research proposes majorly in liver cancer. They are broadly classified into viral and non-viral vectors. Figure 2 Illustrates the targeted therapy for Hepatic Cancer with Viral and Non-Viral Vectors.

### Viral Vectors

These are genetically engineered viruses that exploit their natural ability to infect cells. For creating a viral vector for gene delivery, the pathogenic and replication-related viral genes are typically extracted from viral genome & switched for the genetic arrangement. These involves various therapeutic applications such as in hepatic tumour cell and oncolytic cell killing viruses. Table 1 shows the instances of ligand and transcriptional localization employed in eukaryotic viral vectors for targeted cancer therapy, along with advantages and disadvantages. The most common viral vectors used for liver-targeted gene delivery comprise those based on adeno-associated virus (AAV’s), adenoviruses (Ads), retroviruses (RVs), and lentiviruses (LVs) that are explained below:

**Table 1:** Instances of ligand and transcriptional localization employed in eukaryotic viral vectors for targeted cancer therapy along with advantages and disadvantages^18^, ^19^]

Viral Vectors	Tumor Precise Promoters	Ligand Targeting	Advantages	Disadvantages	References
Adenovirus	GH, TRE, rTG, VEGFR-2,	Homing Peptides: SIGYPLP, CGKRK,	Excellent in vivo and ex vivo transfection efficiency	Strong immune response renders repeated treatments ineffective.	[19, 20]
Adeno-associated virus (AAV)	PRC1, RRM2, BIRC5	NGR, GFE	Good length of expression especially in vivo	Safety issues because of possible insertional mutagenesis.	[21]
Retrovirus	CEA, GRP78, kdr, E-selectin	Not determined	Better transfection efficiency ex vivo	Inefficient transfection in vivo and safety issues	[20]
Lentivirus	PSA/E	αCD20, hSCF	Transfects hemo stem cells and both proliferating and non-proliferating hosts.	Active trafficking into cells, safety issues, and causes of immunodeficiency	[20, 22]

*GH* Growth hormone, *TRE* Tetracyclin response element, *VEGFR2* Vascular endothelial growth factor receptor 2, *rTG* rat thyroglobulin, *SIGYPLP,CGKRK*, *SIKVAV* homing peptides, *PRC1* protein regulator of cytokinesis 1, *RRM2* ribonucleotide reductase subunit 2, *BIRC5* Baculoviral IAP repeat-containing 5, *PSA/E* Prostate-specific antigen/Enhancer, *hSCF* human stem cell factor, *GRP* Glucose Regulated Protein 78, *CEA* Carcinoembryonic antigen

#### Adenoviral vectors

Adenoviruses (ADs) are complex than AAVs and vectors originated from ADs include a 36 kb linear double-stranded DNA genome that codes for a variety of structural and non-structural genes. Human serotypes 2 and 5 are the most commonly used serotypes of these non-enveloped viruses. ADs provide significant hepatotropism, high levels of genetic factor expression in a broad range of cells, ease of routine manufacturing of high functional titres, having enormous packaging capacity, and minimal genotoxicity which makes it an extremely appealing option for hepatic gene delivery [15]. Third-generation AD vectors, also known as Helper-Dependent Adenoviruses (HDAd), are created to achieve long-term expression.

#### Retroviral and lentiviral vectors

Retroviral and lentiviral vectors are coined as powerful tools that are used in gene delivery, especially in gene therapy and biomedical research. These both are derived from the retroviruses (RNA-based viruses that integrate into genetic material into the host genome but differs in targeting tumour cells). These viral genes are packed in specialized cells and introduced into target tumour cells. Retroviral vectors are commonly used to modify the stem cells and other rapidly dividing cells. As it primarily infects the dividing cells (hepatocytes), majorly the reverse transcribed DNA of viral RNA that further integrated into host genome [16]. On the other hand, the lentiviral vectors are subtype of retroviruses (commonly derived from HIV) have the potential to target both dividing and non-dividing cells (such as neurons and muscle cells) due to which these are coined to be more versatile for gene delivery [17]. 

### Non-viral vectors

The nonviral vectors are synthetic or naturally derived systems that are used for gene delivery without using viruses. These vectors are mainly made up of lipid or polymeric elements that make genetic element to enter cells easier. These elements are further used for encapsulation, which help to shield it from inside [19]. Additionally, these vectors provide a remarkable benefit, including easier scalability, a longer shelf life, more potential towards gene delivery, and an increased safety profile as compared to viral vectors. These include liposomes, peptides, inorganic nanoparticles, and dendrimers that are explained below:

#### Liposomes

Conventional liposomes are tailored for specific delivery of macromolecules but they exposed low and inconsistent gene transfer efficiency. The efficiency of gene transfer was revolutionized by the creation of cationic lipids [20]. As a result, a novel delivery mechanism utilizing liposome-DNA complexes, or lipoplexes, were created. Previously, DNA was integrated into liposomes, which makes it possible for negatively charged DNA and positively charged cationic liposomes to interact electrostatically. While the cytotoxicity of these lipoplexes has decreased due to the availability of many cationic lipids with enhanced transfection effectiveness. Additionally, the dioleylphosphatidyl ethanolamine (V-DOPE) is a neutral lipid, which help to release DNA from the endosomes. As a result of this discovery, a combination of DOPE and cationic lipids was used for lipofection followed for gene delivery [21].

#### Peptides and inorganic nanoparticles

Certain peptides and inorganic nanoparticles are also employed as vectors in addition to lipids and polymers. These peptide vectors are short chains of 10–20 residues made up of condensed amino acids such as Trans-Golgi Network (TGN) peptide and synthetic peptides, which are typically composed of positively charged amino acids like arginine or lysine that were demonstrated to be having effective role in condensing DNA [22]. Further, the inorganic nanoparticles are novel non-viral vectors, which are easier to manufacture. These nanoparticles have more potential to pass through cell membranes and enter cells to deliver nucleic acids to the target sites.

#### Dendrimers

Dendrimers are self-assembling nanoscale molecules that are composed of three shells, an inner shell, a symmetric center, and an outer shell. They have low cytotoxicity, more electrostatic interactions, polyvalency, chemical stability, water solubility, and more simplicity of surface modification, which make them appropriate carriers for (Short-interfering- Ribonucleic acid) si-RNA administration [23] Table 2.

**Table 2:** Comparative characteristics, safety considerations, and clinical limitations of viraland non-viral vectors

Category of vectors	Transfection / transduction efficiency	Advantages	Off-target effects and toxicity	Duration of gene expression	Long-term safety considerations	Examples	References
Viral vectors	Generally high; commonly > 50–90% transduction can be achieved in susceptible target cells depending on vector type, dose, and cell type	High gene-transfer efficiency; efficient intracellular delivery; some vectors provide prolonged or stable expression; availability of tissue/cell-specific tropism and engineered targeting; well-established clinical manufacturing platforms	Dose-dependent inflammatory responses and innate/adaptive immune activation may occur. Off-target transduction can cause expression in non-target cells. Pre-existing immunity is particularly relevant for adenoviral and AAV vectors.	**AAV**: months to years, predominantly episomal expression; adenovirus: usually days to weeks; lentivirus/retrovirus: potentially long-term/stable expression following genomic integration	Integration-competent vectors such as retroviral and lentiviral vectors carry a risk of insertional mutagenesis. AAV is generally non-integrating but rare genomic integration and long-term immune/toxicity concerns require monitoring. Persistent vector genomes may also complicate repeat dosing	Adeno-associated virus (AAV), adenovirus, retrovirus, lentivirus	[24]
Non-viral vectors	Generally lower in vivo; often < 10–50% depending on formulation, target tissue, administration route, and delivery system; some optimized lipid nanoparticles can achieve substantially higher delivery in selected tissues	Relatively simple and scalable manufacturing; low risk of insertional mutagenesis; generally lower immunogenicity than many viral vectors; flexible cargo/loading capacity; physicochemical properties can be modified for targeting and controlled release; suitable for repeated administration	Toxicity may arise from cationic lipids/polymers, nanoparticles, or degradation products. Potential off-target uptake by liver, spleen, kidney, and other organs may occur after systemic administration. Complement activation and inflammatory responses have been reported for some formulations	Usually transient, ranging from hours to several days or weeks, although optimized systems can prolong intracellular persistence and expression	Lower risk of genomic integration compared with integrating viral vectors; however, long-term accumulation, biodegradation, organ toxicity, repeated-dose safety, and chronic inflammatory effects require evaluation. Clinical experience remains less extensive for many emerging nanocarriers	Liposomes, lipid nanoparticles, polymeric nanoparticles, peptides, dendrimers, inorganic nanoparticles	[25]

The gene transfer and efficient targeting of mammalian cells of the bacteriophages require a genetic engineering or surface modification. They are genetically flexible and can express targeting ligands, thus they are prospective experimental material for targeted gene delivery, but can also be a limitation due to their intracellular trafficking and clearance by immune cells [6, 24].

## Mechanism of bacteriophage-mediated gene delivery

The engineered bacteriophage-mediated gene delivery to HCC cells is a sequential process of tumor-cell recognition, the internalization, internal trafficking, endosomal escape, therapeutic genetic cargo release, and the expression of the therapeutic genes. The display of the ligands on the surface of the phages may help to facilitate the recognition of receptors expressed on the surface of HCC cells and facilitate the uptake of the receptor-ligand complex [24, 25]. Once internalised, the modified phage need to escape the endosomes and deliver its therapeutic cargo or its DNA to the nucleus where it can be expressed and turn on the targeted anti-cancer pathway. Inefficient endosomal escape, degradation within the cell, and immune clearance, however, are significant challenges in the successful delivery of a phage [26].

### Phage intracellular activity

**Fig. 3:**
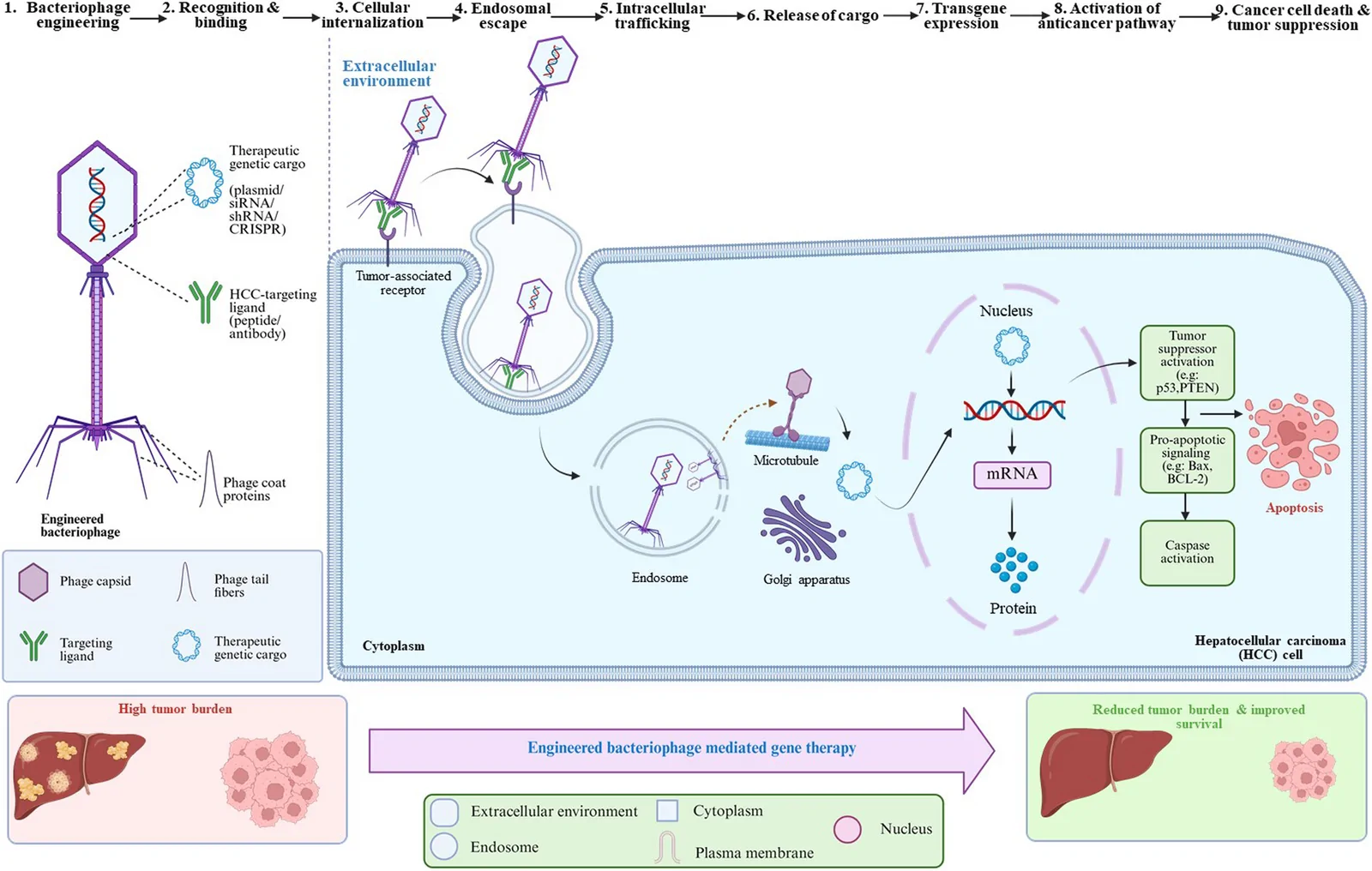
Complete workflow of engineered bacteriophage-mediated gene therapy in Hepatocellular Carcinoma (HCC)

Bacteriophages face unfavourable environmental conditions inside eukaryotic cells, which severely restrict their ability to function in the cytoplasmic space. Similar to certain eukaryotic viruses, phage particles that enter the cell are separated into phagosomes and endosomes, where they are further broken down. Lysosomal protease activity is likely the primary cause of viral particle destruction because bacteriophages are resilient in the severe acidic pH environment of late endosomes and phagolysosomes [26]. Engineered bacteriophages can aid in the delivery of genes to HCC through a sequential process of receptor recognition, internalization, endosomal trafficking, endosomal escape, delivery of therapeutic DNA, nuclear transport and subsequent gene expression [27]. Targeting peptides on the cell surface may facilitate the recognition of tumour-associated receptors and efficient endosomal delivery and escape of therapeutic genes [28, 29] as illustrated in Fig. 3. In addition, phage mammalian cell interactions can also trigger innate immune sensing, such as the RIG-I and cGAS-STING pathways, which can foster antitumor immune activity, but can also promote immune-mediated clearance of the phages. Phage display may consequently be used to discover targeting ligands for tumor-associated molecules like GPC3 and other receptors, thus enhancing the tumor localization and minimizing of the non-specific exposure [29–31].

Although the evolutionary mechanisms by which eukaryotic viruses release their genetic material have been thoroughly studied, whether they involve the blend of the viral membrane with the host endosomal membrane in the case of enveloped viruses or the breakdown & permeability of the endosomal membrane in the case of non-enclosed viruses, the factors which ensure the endosomal escape of phages remain unresolved [32, 33]. Bacteriophage genetic material is thought to be able to bind with cytosolic proteins, helping to activate the RIG-I: (Retinoic Acid-Inducible Gene-I) cGAS-STING: (Cyclic GMP-AMP synthase; STING- stimulator of interferon genes) pathways.

### Phage display technology

The creation and advancement of numerous cancer gene therapy strategies have been greatly aided by bacteriophages. The invention of phage display technology by George Smith in 1985 is among the most significant contributors to the growth of this field of medical oncology [34]. An innovative generation of diagnostic and immunotherapeutic medications is currently being developed using this method, which is also frequently utilized to investigate protein–protein, protein–peptide, and DNA–protein interactions. The insertion of foreign nucleotide arrangements in one of the genes encoding bacteriophage capsid proteins is the basis for this technology. As a result, a diverse range of phage particles are created, each of which displays a unique peptide that is encoded by the integrated DNA fragment. Peptides and their molecular targets are identified by screening the phage display libraries that are so produced [35].

**Fig. 4:**
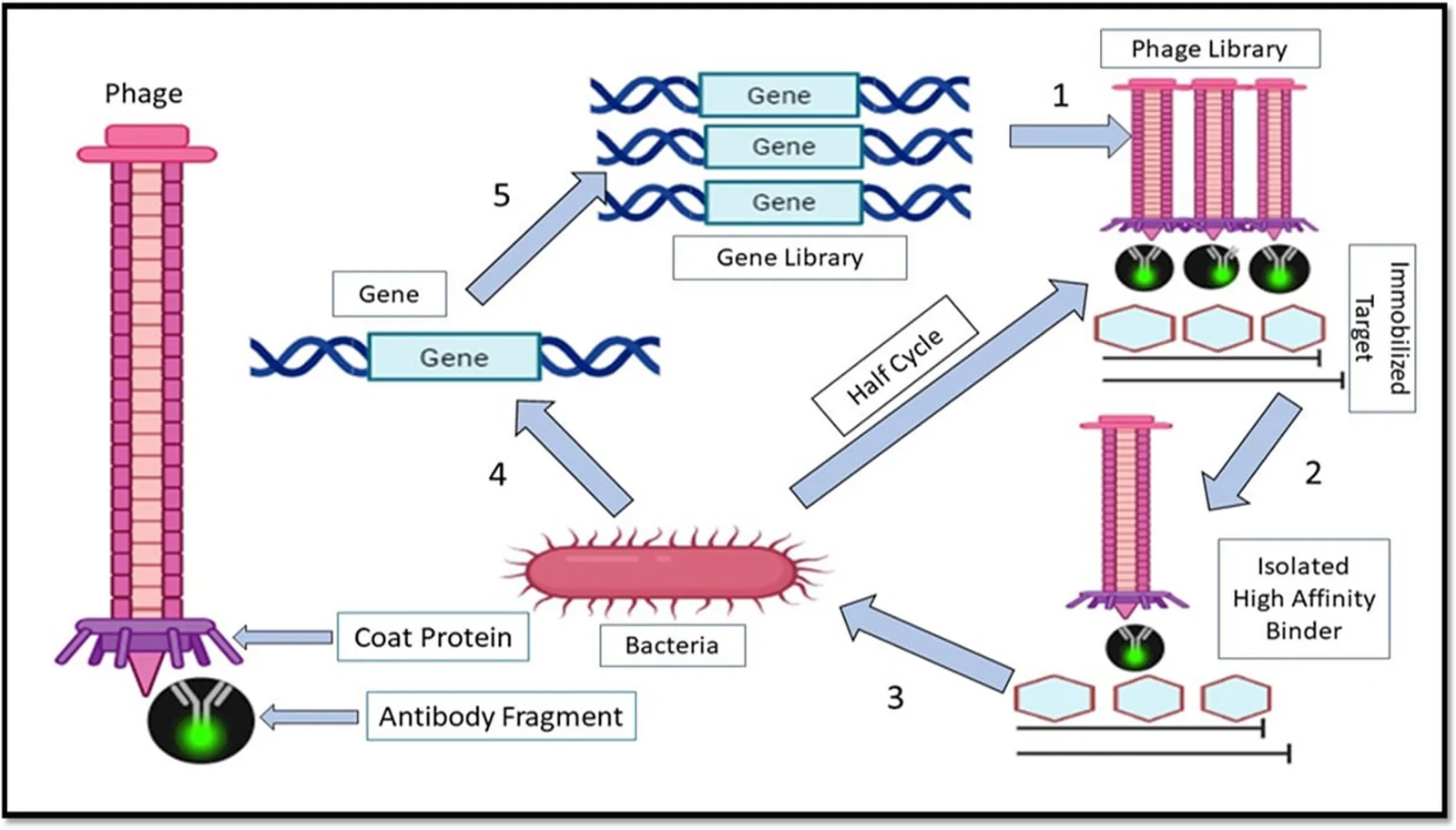
Phage display cycle which occurs through following steps. 1. Fusion proteins for a viral coat protein + the gene to be evolved (typically an antibody fragment) are expressed in bacteriophage. 2. The library of phage is washed over an immobilised target. 3. The remaining high-affinity binders are used to infect bacteria. 4. The genes encoding the high-affinity binders are isolated. 5. Those genes may have random mutations introduced and used to perform another round of evolution. The selection and amplification steps can be performed multiple times at greater stringency to isolate higher-affinity binders

Several characteristics of filamentous Ff phage’s make the development of the phage display possible: initially these viruses reproduce without causing the host cell to lyse that allows for the production of high titers of the phage. Followed by phage capsid proteins are genetically fused with other peptide arrangements, by exposing the foreign protein on the virus surface. In this way, the well-established (phenotype)genotype allows for the selection of a particular phage from large libraries, followed by the identification of the prime sequence of the peptide and to encode it as illustrated in Fig. 4. These days, bacteriophages like T4, T7, and lambda phage are employed for phage display in addition to the filamentous M13 phage [36].

Phage-display technique can screen bioactive peptides with the sequence CRWYDDENAC (RWY) that can bind to the relevant receptors, such as TJ12P1 and L5-peptide-targeting GPC-3 and bioactive peptides targeting integrin. High affinity peptides, like the arginine-glycine-aspartic acid (RGD) peptide [37], which is present in an extracellular matrix and selectively binds to integrin receptors expressed on cell surfaces to mediate adhesion, can also be found thru natural screening in addition to phage-display technology [38]. The RGD peptide can be one of the targets for early targeting since these receptors are also implicated in the development and spread of malignancies. The choice of ligands that can bind to the target selectively is essential for locating the targets on tumor cells in order to accomplish the goal of targeted imaging and treatment. Among these, antibodies such as anti-AFP, anti-GPC-3, and anti-VEGF/VEGFR antibodies are frequently employed in molecular imaging due to their high specificity and targeting, which is a conventional targeting [38]. These investigations showed the high absorption rate of cells and the high concentration aggregation of antibody-modified molecular probes in the tumor region, and subsequent targeted imaging produced outstanding picture quality.

## Application in hepatic cancer

### Targeted delivery of TRAIL gene for HCC

A potential therapeutic molecule is the tumor necrosis factor related apoptosis inducing ligand (TRAIL), which can trigger programmed cell death in cancer cells by activating death receptors [39–41]. The ability of TRAIL to cause cell demise in a very selective way enables it to target cancer cells while preserving healthy cells. The undesirable side effects that are usually connected to conventional cancer treatments are lessened by this selectivity [39]. A comparatively short half-life and quick removal from the bloodstream are two major drawbacks of employing TRAIL to treat cancer, which restricts its ability to reach tumors at therapeutic levels. The safety and effectiveness of TRAIL gene treatment in patients with advanced stages have been assessed in several trials.

Numerous researches have previously documented the use of the inoffensive and non-pathogenic filamentous M13 bacteriophage (phage) as a vector to transfer therapeutic genes in order to target tumors with Transmorphic Phage/AAV (TPA) particles. The particle is designed to include the human adeno-associated virus (AAV2) inverted terminal repeats (ITRs), which are essential for this delivery system, on each side of mammalian gene cassettes [42]. A phagemid-based approach is used to eliminate all phage genes from TPA while keeping the phage replication origin, f1 ori, so that bacteria could replicate the single-stranded TPA DNA and store it using the phage coat proteins supplied by a helper phage infection hepatocellular carcinoma, is a serious worldwide public health issue that varies in incidence by geographic location. Most importantly, in a normal liver cell culture, no TRAIL transgene transfer is found. HCC cells then undergo apoptosis as a result of the TRAIL transgene being produced by the cancer itself. Cellular uptake of engineered M13/AAVP vectors that target cell surface receptors found on HCC cells can be used to deliver the TRAIL gene that will be expressed within the cells and trigger the activation of death-receptor mediated apoptosis via caspase-8 and downstream effector caspases [43]. TRAIL signaling also has the potential to activate the mitochondrial apoptotic pathway through the cleavage of Bid and the activation of cytochrome-c-dependent signaling, and gene delivery can circumvent the limitation of short half-life and poor tumor accumulation of systemically administered recombinant TRAIL protein [44].

### Description of CRISPR/Cas 9 development for hepatic cancer

**Fig. 5:**
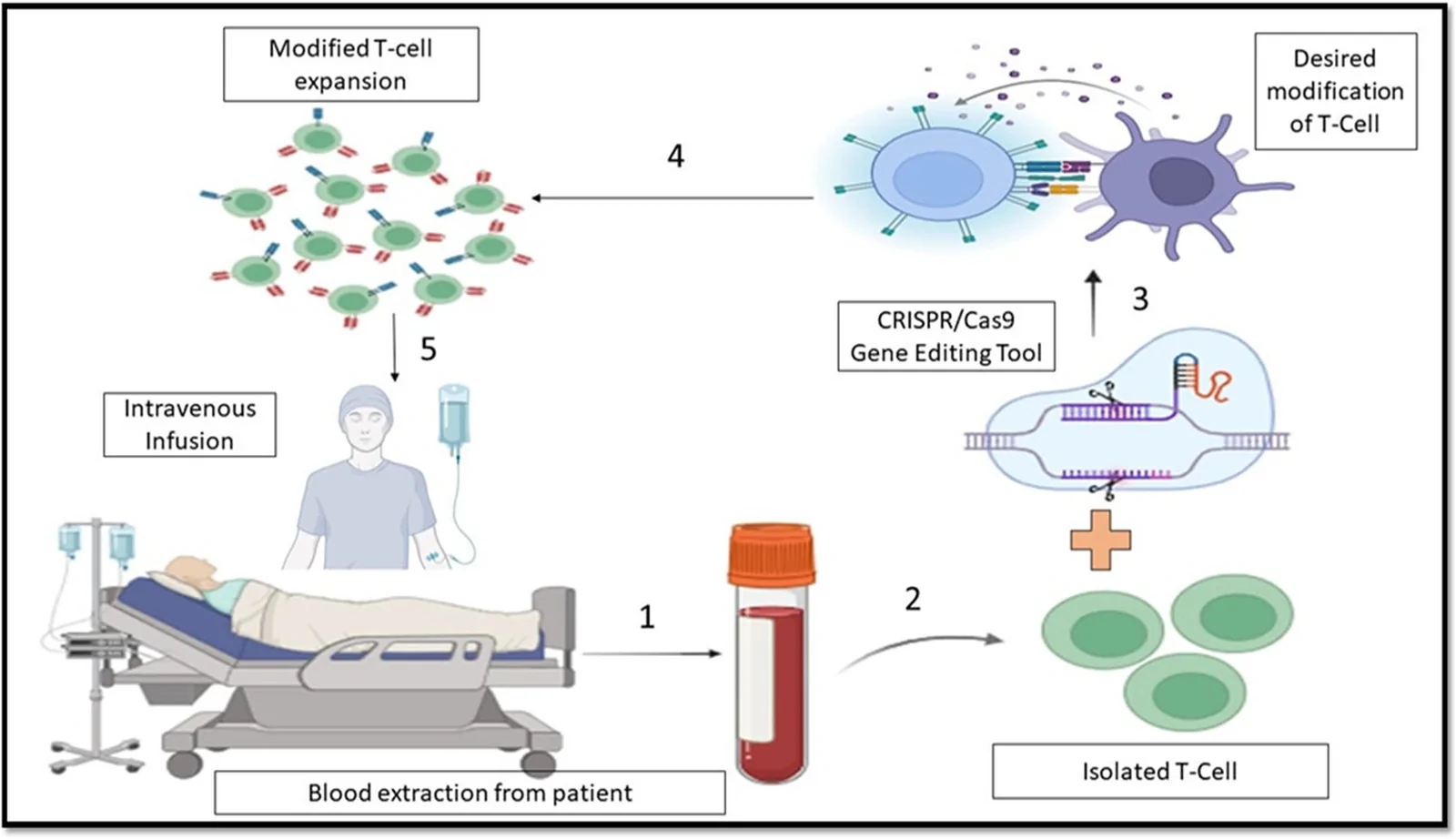
CRISPR/Cas9-mediated Gene Delivery: 1. Desired amount of blood is extracted from patient 2. With the help of isolated T-Cell and CRISPR/Cas9 Gene Editing Tool, 3. T-cell get modified, 4. After that, expansion of modified T-cell occurs, 5. Intravenous infusion of modified T-cell inside the human body

Clustered regularly interspaced short palindromic repeats (CRISPR/Cas-9) is an advanced immune system found in microbes, is the source of the clustered regularly interspaced short palindromic repeat/CRISPR-associated nuclease 9 (CRISPR/Cas9) system [45]. Following modification, it turns into a flexible technology that progressively substitutes earlier gene editing instruments because of its practical, affordable design and wide range of uses as depicted in Fig. 5.

CRISPR creates an RNA-mediated nuclease to cleave the foreign gene and incorporates the fragment of the exogenous gene into a palindromic repeat. Following more investigation, the sequences encoding CRISPR-related proteins could be categorized into five or six groups [46]. For the first time, CRISPR/Cas9 was used as a practical gene editing technique in 2012. Single-guide RNA (sgRNA), a single-stranded RNA that could distinguish target genes and trigger the Cas9 enzyme to cut double-stranded DNA, is created by successfully integrating the double-stranded complex of tracrRNA and crRNA.

The initial step is to incorporate the foreign gene into the CRISPR array. Following phage injection into the host, the foreign gene is broken down into several DNA fragments known as protospacers. The proteins Cas1, Cas2, and Csn2, which are widely distributed in the CRISPR system, must be involved in this. To create a CRISPR array, the protospacers would be chosen and included as new spacers surrounded by repetitive sequences. Protospacer adjacent motifs (PAMs), which are brief sequences found next to target sequences in the external genome, are primarily responsible for protospacer selection [47]. PAMs are identified as a signal of non-self-gene arrangement and are unique to every CRISPR/Cas subtype. The trans-activating CRISPR RNA (tracrRNA) sequence, multiple Cas genes, the leader sequence, and a CRISPR array make up a typical CRISPR locus. RNase III and other nucleases break down the mature tracrRNA-crRNA complex that is created when the pre-crRNA joins tracrRNA. Together with the Cas9 protein, this complex would subsequently recognize and cleave the exogenous gene. Lastly, the CRISPR/Cas9 system would restrict the exogenous DNA from invading. TracrRNA and crRNA work together to activate nucleases through the Cas9 protein. Double-stranded DNA would be cleaved at 3 nt upstream of the PAMs, and the 20-nt crRNA would use the spacer sequence to detect a specific target sequence after the PAMs are located [48].

## Clinical trials

The development of molecular biology and methods like single cell analyses and new generation sequencing, along with traditional diagnostic approaches like computed tomography and biopsies, have greatly advanced our knowledge of the pathophysiology of liver cancer and offered new treatment alternatives. Gene therapy has advanced to the point that it is currently undergoing a number of clinical trials [49] and is now a viable therapy option for a wide range of tumor as described in Table (3 and 4).

**Table 3:** Overview of ongoing clinical trials for gene therapy of hepatic cancers

NCT Number	Types of Liver Tumors	Gene/Antigen	Vectors	Intervention	Route of Administration	Phase	Current Status and Outcomes
NCT00844623	HCC	HSVtk	Adenoviral vector	TK99UN (adenoviral vector containing TK)	Intratumoral injection	1	Accomplished; Outcomes partly reported.
NCT03480152	Primary Liver Cancers	mRNA containing epitopes from immunogenic neoantigens	mRNA vaccine	NCI-4650, a mRNA-based, Personalized Cancer Vaccine	Intramuscular injection	1/2	Terminated; Related results partly reported
NCT01967823	HCC Metastatic Liver Tumors	NY-ESO-1	Anti-NY ESO-1 Murine TCR-Gene Engineered Lymphocytes	Anti-NY ESO-1 mTCR PBL Cyclophosphamide Fludarabine Aldesleukin	Intravenous infusion	2	Recruiting No results available Estimated Completion Date: July, 2028
NCT03198546	HCC	GPC3	CAR-T cells	GPC3 targeting CAR-T cells	Systemic or local injections	1	Recruiting No results available Estimated Completion Date: Aug, 2022
NCT03313596	HCC	HSVtk	Adenoviral vector	ADV-TK LT	Intravenous infusion	3	Recruiting No results available Estimated Completion Date: Dec, 2019
NCT00093548	HCC	AFP, GM-CSF	Adenoviral vector	Vaccination AFP plasmid DNA vaccine GM-CSF plasmid DNA hepatocellular carcinoma vaccine adjuvant	Intramuscular injection/Intradermal injection	1/2	Withdrawn

*HCC* hepatocellular carcinoma, *HSVtk* thymidine kinase of herpes simplex virus, *NY-ESO-1* New York esophageal squamous cell carcinoma 1, *GPC-3* Glypican-3, *LT* liver transplantation

**Table 4:** Critical appraisal of clinical studies evaluating phage-based therapeutic and vector approaches

Phage-based approach	Clinical phase	Indication / Target	Primary objective	Major clinical findings	Safety Profile and Toxicity	Ethical status	Key limitations	Translational significance	Ref
Phage-based therapeutic approach	Phase I/II or early clinical study	Specify disease/target	Primarily safety, tolerability and preliminary efficacy	Clinical benefit should be interpreted according to the predefined endpoint; distinguish statistically significant efficacy from exploratory observations	Report adverse events, immune reactions, dose-limiting toxicity and treatment-related events	Provide ClinicalTrials.gov/WHO ICTRP/EU CTIS identifier and ethics/IRB approval where reported	Small sample size, short follow-up, absence of control group or limited pharmacokinetic/biodistribution data where applicable	Provides preliminary evidence of human feasibility but does not establish clinical efficacy	[48, 49]
Engineered phage/phage-vector system	Early-phase clinical study	Specify target tissue/cell	Safety, delivery efficiency and/or target engagement	Assess whether the vector achieved the intended biological target and whether this translated into a therapeutic response	Evaluate systemic inflammation, immune recognition, off-target effects and treatment-related toxicity	Trial registration and ethics approval should be explicitly reported	Limited human data, uncertain biodistribution, potential immune clearance and limited long-term follow-up	Demonstrates translational potential but requires larger controlled studies	[50]
Phage-derived delivery platform	Phase I/II	Specify disease	Dose escalation, tolerability and preliminary efficacy	Report dose-response relationship and whether efficacy exceeded that of standard treatment/control	Summarize frequency and severity of adverse events	Registration number and ethical oversight	Dose optimization, manufacturing consistency and patient-to-patient variability may limit interpretation	Supports further clinical development if safety and biological activity are demonstrated	[51]
Phage-mediated targeted delivery	Preclinical/first-in-human, if applicable	Specific receptor/cell/tissue	Targeted delivery and therapeutic activity	Human evidence remains limited; preclinical efficacy should not be extrapolated directly to clinical effectiveness	Potential off-target distribution, immune recognition and toxicity require evaluation	Clearly distinguish registered human trials from animal/in vitro studies	Limited clinical experience and insufficient long-term safety data	Promising experimental strategy requiring validation in appropriately powered clinical trials	[52]

## Challenges and limitations of bacteriophages

### Barriers to effective phage-mediated gene delivery

Understanding the barriers to therapeutic intervention and creating strategies to get over them are crucial if we are to realize the full assurance of gene delivery, such as a long-term therapeutic effect or, ideally, a cure. The efficiency of cancer-related gene therapy depends on identifying the appropriate therapeutic gene or genes that can stop the progression of the disease, delivering therapeutic genes to damaged tissue, and achieving the best transgenic expression for inhibiting a gene associated with cancer [50].

Therapeutic genes must be delivered to specific targets in order for a treatment to be effective. Thus, the goal of choosing an appropriate vector for therapeutic gene delivery is essential. Every distribution method, whether viral or non-viral, has its share of challenges. One significant problem with viral vectors, such as adenoviruses, is that humans may already be immune to particular serotypes due to immunizations or natural infections. The use of a heterologous prime-boosting procedures, which involves delivering comparable antigens via several vectors, is one potential strategy to overcome this difficulty [51].

### Scalability, formulation and regulatory hurdles

The lack of uniform regulatory frameworks and the requirement for unique approval for every bacterial strain present challenge for bacteriophage-based gene delivery [52]. The creation of adaptable, flexible regulatory mechanisms that are specific to phage therapy is necessary to meet these problems. The preservation of purity, stability, and consistency throughout the large-scale manufacture of phages can be difficult and expensive. Technological developments in biomanufacturing are crucial to resolving these problems because they allow for more accurate and efficient production procedures.

Immune clearance and poor systemic persistence are two significant limitations. The mononuclear phagocyte system quickly identifies and eliminates phages after intravenous injection, especially in the liver and spleen, resulting in significantly shorter half-lives [53]. Neutralizing antibodies are frequently triggered by repeated dosage, which further reduces bioavailability. In oncology, where a sustained systemic presence is necessary for sufficient tumor penetration, this problem is especially pertinent. Surface alterations like PEGylation have become useful countermeasures: PEG chains prolong circulation durations and decrease immune detection by sterically shielding capsid epitopes [54]. Although other formulations, including as liposomal encapsulation and nanoparticle conjugation, can further increase intratumoral transport and stability, striking a balance between infectivity and immune evasion is still a challenging design trade-off [55]. The limitations of phage therapy’s manufacture and scalability are equally important. Personalized preparations frequently require the time-consuming, resource-intensive, and technically challenging isolation, amplification, and characterisation of patient-specific phages [56]. To get rid of impurities including endotoxins, host cell proteins, and leftover bacterial DNA, Good Manufacturing Practice (GMP) compliance is crucial. However, phage preparations are intrinsically heterogeneous and necessitate custom production pathways, making standardized mass manufacture challenging, in contrast to chemically homogenous antibiotics or monoclonal antibodies. A lack of widely recognized purification procedures, difficulties sustaining formulation stability over time, and the logistical strain of creating multi-phage cocktails for customized therapies are other barriers [57]. These restrictions result in increased expenses and longer lead times, which are especially important for cancer patients with impaired immune systems who need quick infection control. Adoption of phage therapy is hampered by doctors’ and patients’ lack of knowledge about its benefits. Understanding of its advantages and uses can be promoted by implementing focused outreach initiatives and educational campaigns. One major obstacle to the wider application of phage therapy is the lack of established protocols in clinical settings [58]. Significant obstacles to the broad use of individualized phage therapy include its high cost and restricted availability [59]. Costs can be decreased and accessibility enhanced by increasing production and funding research to create economical manufacturing techniques.

## Conclusion and future perspectives

With the development of molecular tools to deliver genes to targeted cells, bacteriophages have appeared as a potential therapeutic and gene delivery platform for cancer. Because of these viruses’ genetic plasticity and simplicity of modification, engineered bacteriophages can now be used as diagnostic and therapeutic tools for a variety of illnesses, including cancer. In comparison to vectors grounded on eukaryotic viruses, the modified phage particles offer a safer and extra precise systemic delivery of therapeutic payload to malignance cells [24]. Innovative vector systems and transform particles that exhibit exceptionally high levels of targeted gene expression in eukaryotic cells are developed as a result of efforts to improve the effectiveness of cancer gene therapy utilizing bacteriophages. For instance, a promising approach for malignancy gene therapy involves merging these next-generation vectors with CRISPR/Cas9 genome editing technologies. The development of controlled nanoparticles with highly selective nuclease activity targeted at eradicating mutant oncogenes could result from future optimization of this technology. Utilizing the distinct advantages of added bacteriophages to produce novel hybrid viruses could increase the variety of treatment approaches, even though the chimeric hybrid vector of adeno-associated virus and phage (AAVP) is being employed for targeted gene treatment for a growing variety of tumors [60]. Further comprehensive study on the molecular mechanisms underlying the interaction of bacteriophages with eukaryotic cells, particularly cancer cells, is likely to uncover novel approaches to enhance phage-mediated transduction.

## Data Availability

No datasets were generated or analysed during the current study.
